# Supplementary material for: Thyroid Hormone Signaling in Embryonic Stem Cells: Crosstalk with the Retinoic Acid Pathway
Source: Int J Mol Sci. 2020 Nov 25;21(23):8945. doi: 10.3390/ijms21238945 (PMC7728128; doi:10.3390/ijms21238945)
Supplement: Supplementary file 1 [file ijms-21-08945-s001.zip › Supplemental material copia/ijms-1001422-Supplemental Figure-corrected-proof.doc]

**Supplemental Figure. Klf9 gene expression after RA and TR antagonist 1-850 treatment.** RA induced a significant increase in the mRNA expression of *Klf9, however n*o significant TR antagonist 1-850 effect was observed. Graphs contain mean values ± SEM from 2-3 independent experiments performed in duplicate. Statistical analysis performed: Student’s t-test. *Abbreviations: Klf9, Krüppel‐like factor 9; RA, retinoic acid; TR, thyroid hormone receptor.*

*Klf9*	Gene bank accession number: NM_057211.1.

Primers:	*Forward*	5’- AGTGGCTTCGAAGGGGAAAC-3’;

*Reverse*	5’- TCCGAGCGCGAGAACTTTT-3’
